# Changes in Hemoglobin Properties in Complex with Glutathione and after Glutathionylation

**DOI:** 10.3390/ijms241713557

**Published:** 2023-08-31

**Authors:** Iuliia D. Kuleshova, Pavel I. Zaripov, Yuri M. Poluektov, Anastasia A. Anashkina, Dmitry N. Kaluzhny, Evgeniia Yu. Parshina, Georgy V. Maksimov, Vladimir A. Mitkevich, Alexander A. Makarov, Irina Yu. Petrushanko

**Affiliations:** 1 Engelhardt Institute of Molecular Biology, Russian Academy of Sciences, Moscow 119991, Russia; julia_kul2000@mail.ru (I.D.K.); waldgang@tuta.io (P.I.Z.); yuripoul@gmail.com (Y.M.P.); anastasia.a.anashkina@mail.ru (A.A.A.); uzhny@mail.ru (D.N.K.); mitkevich@gmail.com (V.A.M.); aamakarov@eimb.ru (A.A.M.); 2 Faculty of Biology, Lomonosov Moscow State University, Moscow 119234, Russia; parshinae@gmail.com (E.Y.P.); gmaksimov@mail.ru (G.V.M.)

**Keywords:** hemoglobin, glutathione, glutathionylation, circular dichroism, Raman scattering, infrared spectroscopy, tryptophan fluorescence, differential scanning fluorometry, molecular modeling

## Abstract

Hemoglobin is the main protein of red blood cells that provides oxygen transport to all cells of the human body. The ability of hemoglobin to bind the main low-molecular-weight thiol of the cell glutathione, both covalently and noncovalently, is not only an important part of the antioxidant protection of red blood cells, but also affects its affinity for oxygen in both cases. In this study, the properties of oxyhemoglobin in complex with reduced glutathione (GSH) and properties of glutathionylated hemoglobin bound to glutathione via an SS bond were characterized. For this purpose, the methods of circular dichroism, Raman spectroscopy, infrared spectroscopy, tryptophan fluorescence, differential scanning fluorimetry, and molecular modeling were used. It was found that the glutathionylation of oxyhemoglobin caused changes in the secondary structure of the protein, reducing the alpha helicity, but did not affect the heme environment, tryptophan fluorescence, and the thermostability of the protein. In the noncovalent complex of oxyhemoglobin with reduced glutathione, the secondary structure of hemoglobin remained almost unchanged; however, changes in the heme environment and the microenvironment of tryptophans, as well as a decrease in the protein’s thermal stability, were observed. Thus, the formation of a noncovalent complex of hemoglobin with glutathione makes a more significant effect on the tertiary and quaternary structure of hemoglobin than glutathionylation, which mainly affects the secondary structure of the protein. The obtained data are important for understanding the functioning of glutathionylated hemoglobin, which is a marker of oxidative stress, and hemoglobin in complex with GSH, which appears to deposit GSH and release it during deoxygenation to increase the antioxidant protection of cells.

## 1. Introduction

Red blood cells transport oxygen to body tissues with the help of hemoglobin, an oxygen-binding protein. Hemoglobin is a tetramer which consists of two alpha and two beta subunits, each containing a heme that binds one oxygen molecule. The hemoglobin molecule can be in three different states. The first one, methemoglobin, is characterized by its inability to bind oxygen and the oxidized form of Fe^3+^ in the heme (HbFe^3+^). Oxyhemoglobin, an oxygen-bound form (HbFe^2+^-O_2_), and deoxyhemoglobin, an oxygen-free form (HbFe^2+^), contain iron in the reduced form (Fe^2+^). The structure of hemoglobin was first resolved and described by Perutz in 1970 [1]. Oxygen binding to the four binding sites is cooperative and leads to a conformational transition from the tense state characterized by low oxygen affinity to the relax state characterized by high oxygen affinity.

The hemoglobin concentration in erythrocytes is very high at about 5.5 mM, which is close to the solubility limit of this protein. Red blood cells have a limited supply of proteins, as they cannot synthesize proteins and are constantly exposed to various stress factors; therefore, a well-functioning antioxidant defense system appears to be crucial for this cell type. The significant part of this system is glutathione, the main low-molecular-weight thiol of erythrocytes, which protects proteins from irreversible oxidation. The redox potential of the reduced glutathione (GSH)/oxidized glutathione (GSSG) pair determines the redox potential of the cell [2]. The concentration of reduced glutathione is about 5 mM, which is close to the concentration of hemoglobin. We showed for the first time the existence of a noncovalent complex of hemoglobin with GSH [3]. The isothermal titration calorimetry revealed that hemoglobin in the oxy form binds four GSH molecules, while the deoxy form binds only two GSH molecules, evidentially, explaining the rising levels of glutathione during hypoxia both in human erythrocytes at altitude and in isolated erythrocytes under hypoxic conditions [3]. Thus, hemoglobin can act as a depot for GSH, releasing it in a hypoxic environment, which probably plays a significant role in enhancing the antioxidant defense of erythrocytes, protecting cells from reactive oxygen species produced by mitochondria in peripheral tissues sensitive to drastic changes in oxygen levels [3]. In addition, it was shown that the formation of a noncovalent complex of hemoglobin with GSH leads to a slight increase in the affinity of hemoglobin for oxygen [3]. The effect of complex formation with GSH on hemoglobin structure has not been described yet.

Glutathione is also able to covalently bind to the thiol groups of proteins, leading to glutathionylation, i.e., to the formation of an SS bridge between the glutathione molecule and the protein molecule. S-glutathionylation can be realized through different mechanisms [4], one of which is thiol–disulfide exchange between protein thiol groups and GSSG. During oxidative stress, part of the reduced glutathione is consumed to neutralize active radicals, resulting in a GSH level decrease and a GSSG level increase, which induces the glutathionylation of proteins [4]. Thus, glutathionylation protects the thiol groups of proteins from irreversible oxidation [4,5]. S-glutathionylation can be reversed by enzymes [4,5]. When the redox status is normalized, protein deglutathionylation occurs [4]. The glutathionylation of hemoglobin is considered to be a marker of oxidative stress of erythrocytes [6]. In addition to physiological responses to oxidative stress, glutathionylation appears in various pathological conditions, such as diabetes mellitus, chronic renal failure, iron deficiency anemia, hyperlipidemia, Friedreich’s ataxia, atherosclerosis, and chronic renal failure [6,7,8]. We have shown that in addition to oxidative stress, glutathionylation can also be caused by a depletion of energy resources of erythrocytes that occur during hypoglycemia [9]. Glutathionylation has not only a defense function, protecting the thiol groups of hemoglobin from irreversible oxidation, but also a regulatory function, significantly (6-fold) increasing the affinity of hemoglobin for oxygen. In order to explain this phenomenon, attempts were made to identify structural changes caused by glutathionylation. In one of the first studies [10], it was shown using NMR that the intra-subunit salt bridge and inter-subunit hydrogen bond at the interface between αβ dimers, which are present in the deoxy form, dissociate during glutathionylation. The similar effect observed in the transition from the deoxy form to the oxy form of hemoglobin provides evidence of the conformational shift of deoxyhemoglobin to an oxyhemoglobin-like conformation. Using isotope exchange kinetics monitored through mass spectrometry, it was found that conformational flexibility was increased in the glutathionylated hemoglobin region, resulting in a loosening of the region [11]. However, the alteration in the secondary structure of glutathionylated hemoglobin has not yet been described and interpreted.

In this study, the effects of the formation of a noncovalent complex of hemoglobin with glutathione and glutathionylation of hemoglobin on the secondary structure of hemoglobin, the heme environment, conformation of the hemoporphyrin macrocycle, tryptophan fluorescence, and protein thermal stability were determined. The data obtained are important for understanding the mechanisms underlying the change in hemoglobin function during its covalent and noncovalent binding to glutathione.

## 2. Results

### 2.1. Evaluation of the Effectiveness of Hemoglobin Glutathionylation during Incubation with Oxidized Glutathione (GSSG)

**Figure 1:**
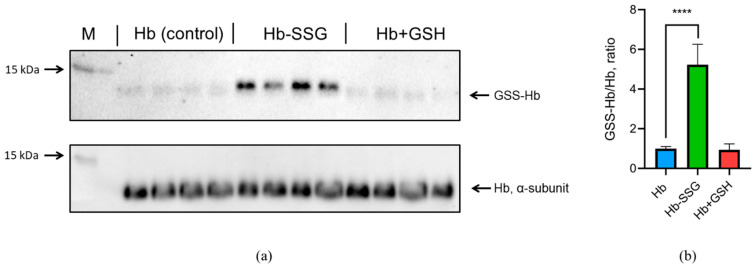
(**a**) The original immunoblotting readouts, mouse monoclonal antiglutathione antibody, and rabbit monoclonal anti-α oxyHb subunit antibody were applied to detect glutathionylated proteins and total amount of α-subunit, respectively. (**b**) Bars represent the S-glutathionylated form of the oxyHb (GSS-Hb) normalized to total amount of the oxyHb. Data are mean values for three independent experiments with triplicates ± standard deviation. n = 4, **** *p* < 0.005.

The oxygen saturation of hemoglobin (SaO_2_—oxygen saturation, %) approaches 100% at an oxygen partial pressure above 17 kPa (128 mmHg) [12]. Therefore, under normal atmospheric conditions with an oxygen partial pressure of about 21 kPa (158 mmHg, at atmospheric pressure of 760 mmHg), almost all purified hemoglobin is in the oxy form (Fe^2+^ with O_2_) [12], which was confirmed for our samples by spectrophotometry (Figure S1, Supplementary). To determine the efficiency of methemoglobin (metHb) reduction with sodium dithionite, we used the first derivative of the spectrum at 645 nm according to the protocol described in [13]. The results are presented in Figure S2 (Supplementary). For hemoglobin, hemoglobin after incubation with GSSG, and hemoglobin after incubation with GSH, the content of metHb is less than 2.4%. Thus, the content of metHb is low and the same in all three studied forms of hemoglobin during our experiment. The efficiency of oxyhemoglobin glutathionylation by GSSG was determined using immunoblotting. It was shown that incubation with GSSG resulted in a significant (more than fivefold) increase in oxyhemoglobin glutathionylation (Figure 1). At the same time, there was no increase in glutathionylation during the incubation of hemoglobin with GSH (Figure 1, Figure S3, Supplementary). This confirms our earlier data that incubation with GSH leads to noncovalent complex formation with oxyHb only [3].

### 2.2. Evaluation of Changes in the Circular Dichroism Spectrum in the Region of the Soret Band during the Glutathionylation of Hb and the Formation of a NonCovalent Complex of Hb with GSH

**Figure 2:**
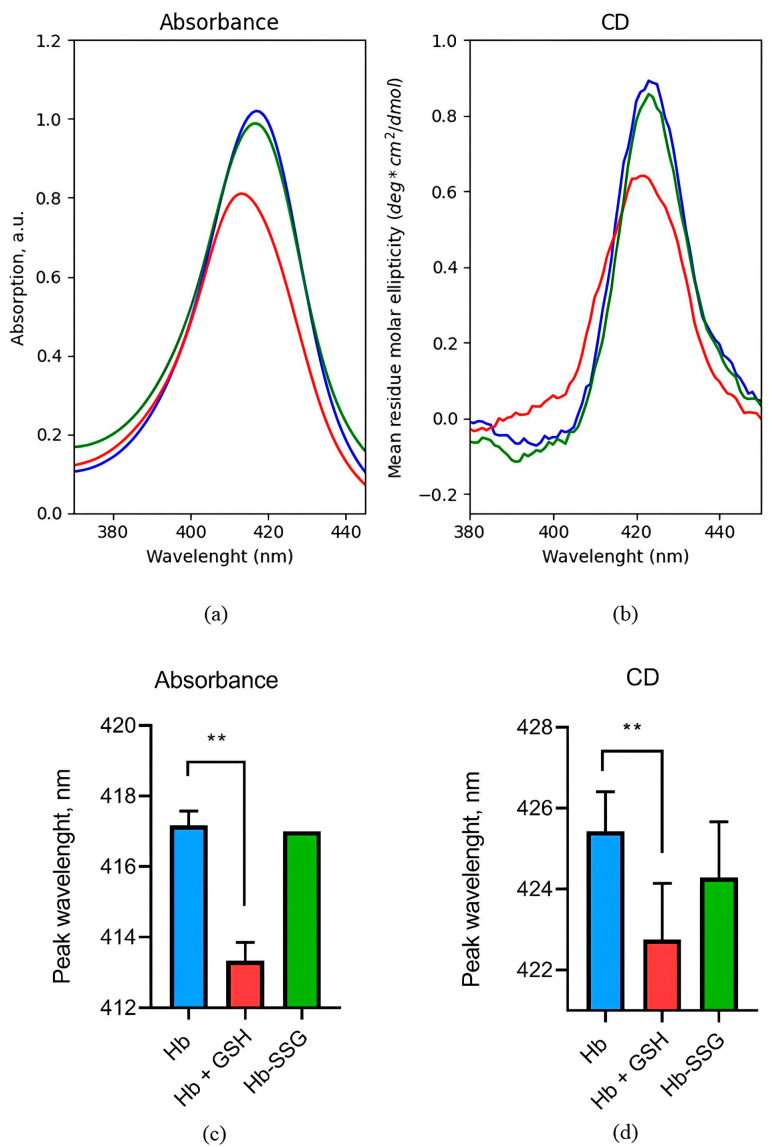
Representative absorption spectrum (**a**) and circular dichroism spectrum (**b**) in the Soret band region for oxyHb (blue), oxyHbSSG (green), and oxyHb+GSH (red). Wavelength values of the absorption spectrum maximum (**c**) and the maximum in the circular dichroism spectrum (**d**) in the Soret band region for oxyHb (blue), glutathionylated hemoglobin—oxyHbSSG (green), and noncovalent complex of hemoglobin with glutathione—oxyHb + GSH (red). n = 3, ** *p* < 0.005.

Absorption and circular dichroism of hemoglobin in the Soret band region are caused by electronic transitions of hemes. It was found that glutathionylation did not affect the hemoglobin circular dichroism (CD) spectrum and the absorption spectrum in the Soret band region (Figure 2), indicating that the position of heme groups and their environment remained unchanged. The formation of a noncovalent complex of hemoglobin with GSH led to a 24% decrease in the amplitude of the circular dichroism peak and a shift in the peak position by 3 nm to the short-wave region (Figure 2). In this case, the amplitude of the absorption peak in the Soret band decreased by 20%, and the peak also shifted by 4 nm to the short-wavelength region (Figure 2), which is associated with the changes in the position of heme groups and their environment during the formation of a noncovalent complex of hemoglobin with GSH.

### 2.3. Evaluation of Secondary Structure Changes during Hemoglobin Glutathionylation and the Formation of Noncovalent Complex Hemoglobin with GSH

**Figure 3:**
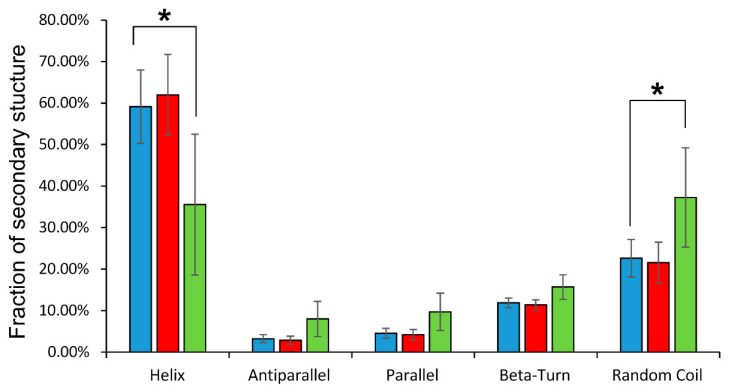
Distribution of secondary structure components of oxyHb (blue), oxyHbSSG (green), and oxyHb + GSH (red) obtained in the CDNN program. On the vertical axis is the percentage of secondary structures; on the horizontal axis are the names of the types of secondary structure defined by the program. Data are presented as mean ± SD, n = 6. * *p* < 0.05.

Circular dichroism spectra in the ultraviolet region reveal general changes in the secondary structure of globin. It was found that glutathionylation decreased in the proportion of alpha-helicity of the protein by almost 25%, increasing the percentage of disordered structures (Figure 3).

The formation of the noncovalent complex of hemoglobin with GSH did not affect the secondary structure of the protein (Figure 3).

### 2.4. Evaluation of Changes in Tryptophan Fluorescence Spectra during Hb Glutathionylation and the Formation of a Complex Hemoglobin with GSH

The intrinsic fluorescence of hemoglobin is due to the presence of tryptophans and tyrosines in protein core [14,15] or heme moiety [16]. The main contribution to the intensity of tryptophan UV fluorescence of hemoglobin is Trp37 located at the interface of two αß dimers [14], and the detected signal is sensitive to changes in the tertiary structure of the protein, as well as to ligand binding [15]. In these series of experiments, the fluorescence spectrum of hemoglobin was recorded at an excitation wavelength of 296 nm, which corresponds to an excitation peak of tryptophans [14].

**Figure 4:**
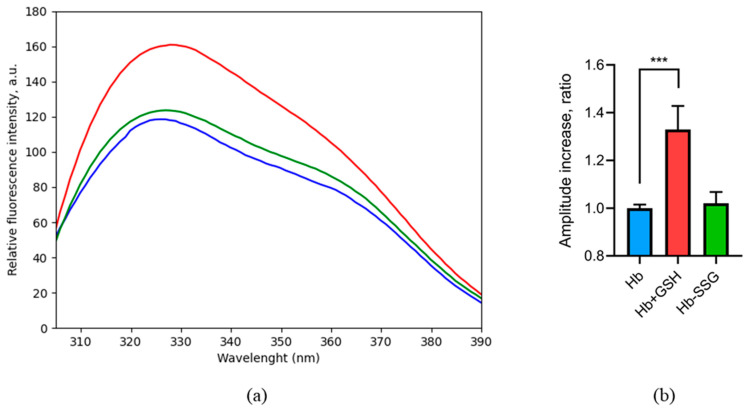
Fluorescence spectrum (**a**) of oxyHb (blue), oxyHbSSG (green), and oxyHb + GSH (red), λex = 296 nm. Change in the peak amplitude (**b**) for oxyHb (blue), oxyHbSSG (green), and oxyHb+GSH (red) as the ratio of the peak amplitudes of the samples to the amplitude of the peak in the control. For the calculation, average values were taken in the wavelength range of 329–333 nm. n = 3, *** *p* = 0.002. (ANOVA).

The intensity of tryptophan fluorescence of hemoglobin remained unchanged after the glutathionylation; consequently, the impact of glutathionylation on the intramolecular positions of tryptophans and the tryptophan environment is negligible (Figure 4).

On the contrary, the formation of a noncovalent complex with GSH increased the intensity of tryptophan fluorescence (Figure 4), which evidentially caused changes in the hemoglobin tryptophan microenvironment, including tryptophan shielding during complex formation and positional shifts of tryptophan residues in the globin molecule when the protein structure changed [15]. Previously, we predicted four noncovalent binding sites of oxyhemoglobin to GSH by molecular modeling [3]. According to the modeling data, Trp37 is included in the third and fourth binding sites. Thus, it is possible that the change in fluorescence is associated with a change in the environment of this residue during the formation of a complex with GSH.

### 2.5. Modeling of Tryptophan Availability

**Figure 5:**
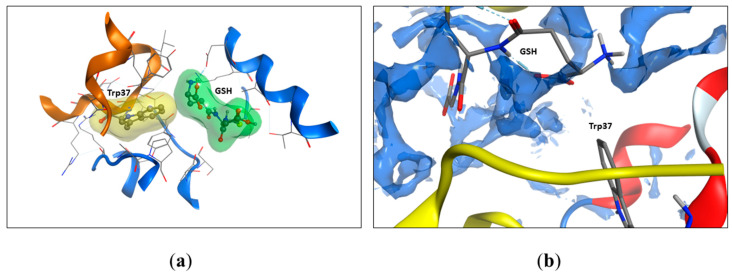
The GSH molecule restricts water access to the Trp37 residue. (**a**) The accessible surface area of Trp37 to the solvent decreases from 54.9 % to 7.8% upon GSH binding (according to Table 1). The molecular surface was built around the GSH molecule (green) and the Trp37 (yellow) residue. Oxyhemoglobin molecules are shown as cartoons, alpha chain in brown, and beta chain in blue. (**b**) The interaction between GSH and Trp37 (beta chain) is hydrophobic. The solvent-accessible regions of the hemoglobin chains after GSH binding are shown as dark blue surfaces. Protein structure of beta chain colored by secondary structure elements – red for α-helixes, alpha chain is shown in yellow. GSH atoms are shown as sticks in standard atom coloring (red for oxygen, gray for carbon, blue for nitrogen).

In the previous work, we found that only Trp37.β was involved in the interaction with glutathione [3]. To evaluate the changes in the accessibility of Trp14.α, Trp15.β, and Trp37.β, we calculated the solvent-accessible surface area (SASA) of oxyHb per residue (Table 1). Thus, we found that noncovalent bound glutathione altered the accessibility of both Trp37.β (from 54.9 to 8.0–7.8%) and did not affect other Trp residues’ accessibility (Table 1, Figure 5). Two of four GSH interaction sites were located near Trp37.β, but in one case, there was an H-bond between Trp37.β2 and GSH; in another, there was no H-bond, but GSH was located near Trp37.β1, isolating it from the solvent. The full output data regarding Trp’s SASA are listed in Table 1.

### 2.6. Effect of Hb Glutathionylation and Formation of Hb Complex with GSH on Protein Thermal Stability

**Figure 6:**
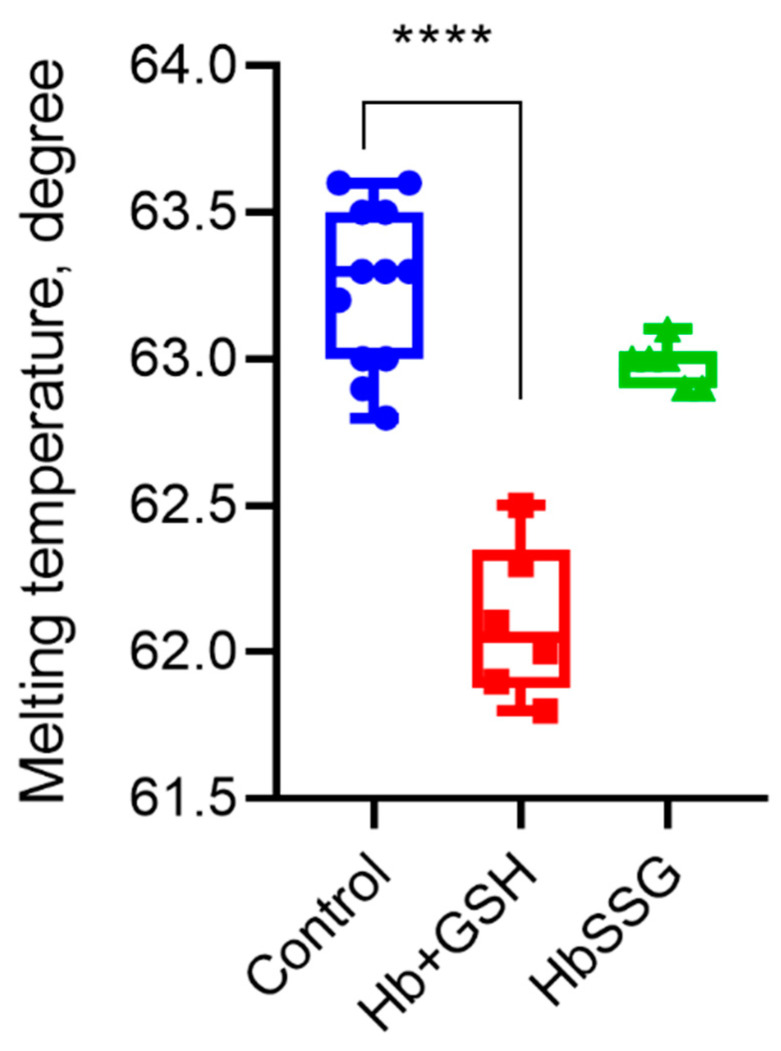
Melting temperature of hemoglobin oxyHb (blue), oxyHb + GSH (red), and oxyHbSSG (green), n = 6, **** *p* < 0.0001.

Using differential scanning fluorimetry, we found that glutathionylation did not change the parameter characterizing the globin structure—the melting point of the protein. The formation of a noncovalent complex of hemoglobin with GSH led to a decrease in the melting point and, consequently, the thermal stability of the protein (Figure 6).

### 2.7. Effect of Hb Glutathionylation and Hb Complex Formation with GSH on the Conformation of the Hemoporphyrin Macrocycle of the Heme of Hemoglobin

**Figure 7:**
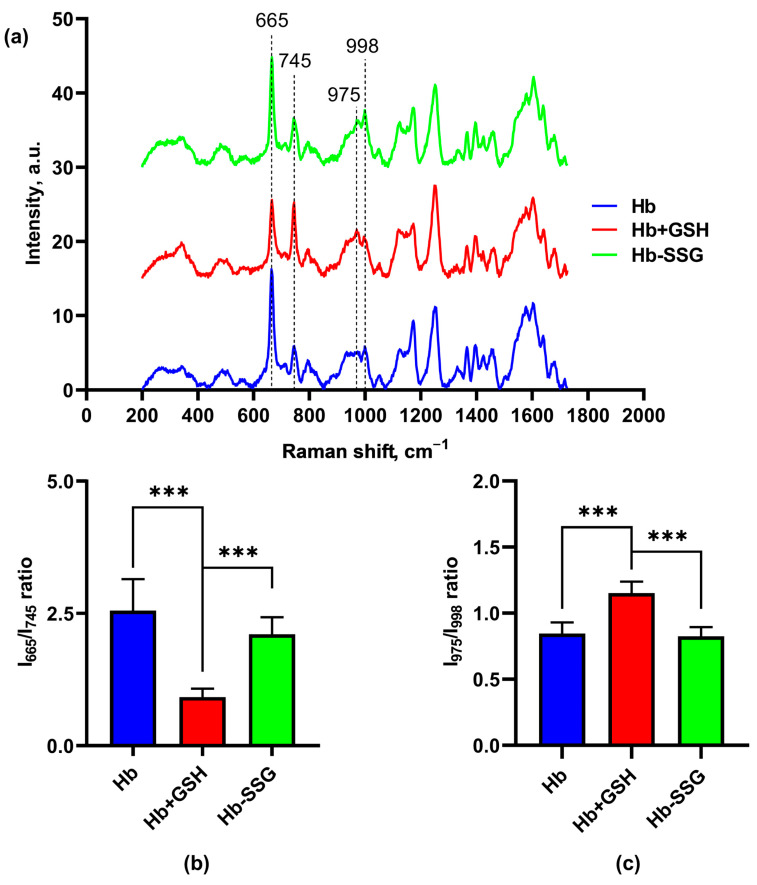
Averaged Raman spectra (**a**) of hemoglobin oxyHb (blue), oxyHb + GSH (red), and oxyHb-SSG (green). (**b**) Intensity ratio of the I (665 cm^−1^)/I (745 cm^−1^) bands in the Raman spectra. (**c**) Intensity ratio of the I (975 cm^−1^)/I (998 cm^−1^) bands in the Raman spectra. n = 8, *** *p* < 0.001.

Figure 7 shows the averaged Raman spectra of dried samples of hemoglobin, Hb-SSG and Hb + GSH. We used oxyHb in the dried state because the Raman signal of oxyHb in the solution was much lower due to nonspecific fluorescence background. In our experiment, we took the edge of the fully dried drop of the sample, so it can be presumed that the amount of water taken for the analysis was negligible. The spectra we obtained from our samples were very close to the spectra from [18]. The authors of this article measured Raman spectra from tiny crystals of Hb, and the spectra we obtained were the same, so we concluded that, in our case, Hb was also in the crystalline form.

The presented Raman spectra do not correspond to the spectrum of hemoglobin in solution, but in crystalline form when Raman is excited by a laser with a wavelength of 633 nm. Crystalline hemoglobin, that exhibits a resonant enhancement of a number of bands under 633 nm laser excitation, significantly affected the shape of the spectrum [18,19].

In these series of experiments with a noncovalent Hb + GSH complex, significant changes in the conformation of the porphyrin macrocycle were observed in the ratio of the bands of the CR spectrum: 665 and 745 cm^−1^ (Figure 7b), which correspond to vibrations of certain bonds of the porphyrin molecule, namely: 665 cm^−1^ (ν_7_)—symmetric deformation vibrations of pyrrole rings, and 745 cm^−1^(ν_15_)—“breathing” vibrations (symmetric stretching vibrations) of pyrrole rings [18].

In the Hb + GSH samples, this ratio was less than in the control and in the Hb-SSG samples. Thus, in the samples with noncovalently bound glutathione, the resonance enhancement for band 665 cm^−1^ was smaller than for the other samples. This effect probably depends on the nature of crystal packing of the protein, since the resonance amplification is characteristic only for the dense packing of molecules in a crystalline sample [18,19].

In addition, differences were observed in the ratio of the 975 and 998 cm^−1^ bands (Figure 7c). The band 975 cm^−1^ (ν_46_) corresponds to asymmetric stretching vibrations of C_b_C_1_ (lateral radical). The change in the conformation of the heme macrocycle is evident from the band 998 cm^−1^ (ν_47_) corresponding to asymmetric deformation vibrations of pyrrole rings. Both of these bands, according to the data of [18,19], resonantly amplified when crystalline hemoglobin was excited with light of 633 nm wavelength. The decreasing ratio of these bands in the Hb + GSH complex relative to the control and Hb-SSG apparently indicates structural changes in the region of the hemoporphyrin side radicals of the hemoglobin molecule.

### 2.8. Effect of Hb Glutathionylation and Hb Complex formation with GSH on the Infrared Spectrum of Hemoglobin

**Figure 8:**
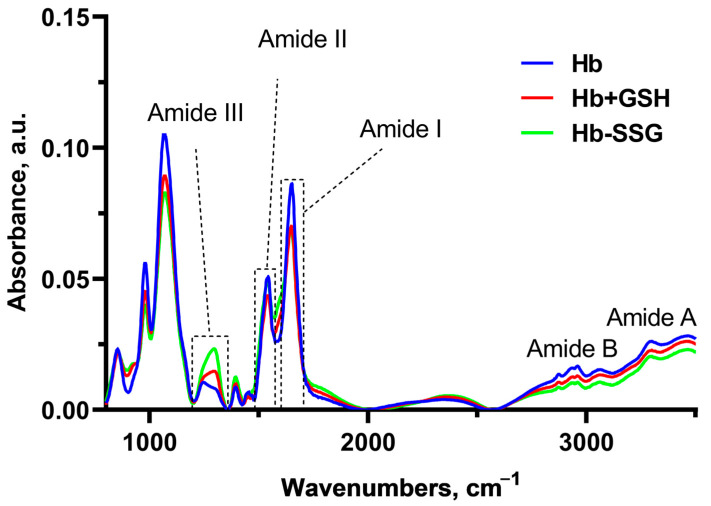
Averaged FTIR spectra of hemoglobin—oxyHb (blue), oxyHb + GSH (red), and oxyHb-SSG (green).

In these series of experiments, Fourier-Transform InfraRed (FTIR) spectra of hemoglobin were recorded (Figure 8) for the study of changes in globin conformation (Figure 8). In this setting of the experiment, infrared (IR) spectrum bands characteristic of Amide I, II, III, A, and B, as well as a group of bands corresponding to vibrations of C-H bonds of side radicals of amino acids, were revealed (2874 cm^−1^–2961 cm^−1^) [20].

Significant changes in the FTIR spectra during the formation of the Hb + GSH complex and Hb-SSG were detected in the region 1650 cm^−1^–1243cm^−1^ (Figure 8). The band at 1650 cm^−1^ corresponds to group vibrations of the amide bond in the protein molecule (Amide I); the greatest contribution to this type of vibrations was made by C=O vibrations. The maximum of the band is characteristic of the α-helical structure [21,22,23]. The 1552 cm^−1^ band corresponds to the group vibrations of peptide bonds of Amide II, including deformational N-H vibrations and valence C-N vibrations [21]. The bands at 1453 cm^−1^ and 1393 cm^−1^ characterize the bending of CH_2_ groups and the symmetric stretching of CH_3_ groups, respectively [22]. In the region of 1244 cm^−1^ –1312 cm^−1^, there was a lower-intensity region corresponding to the group vibrations of Amide III [20,23], which was also associated with bending vibrations of N–H and stretching vibrations of C–N.

It is known that the position of the Amide I band changes with a change in the secondary structure of the protein. Usually, for a protein molecule, the shape of the Amide I band is determined by the superposition of the bands of all secondary structure elements included in the protein. For the alpha helix structure, the maximum usually localizes in the region of 1650 cm^−1^ [24]. Hemoglobin is a globular protein, and alpha helices predominate in its secondary structure; the position of the maximum of Amide I corresponds to the maximum for the alpha-helical structure [25]. A decrease in the relative amplitude of Amide I compared with the amplitude of the Amide II band, which is significantly less dependent on the secondary structure of the protein, may indicate a decrease in the relative content of alpha helices due to an increase in the proportion of other types of secondary structure (beta layers or disordered structure). The ratio of Amide I/Amide II amplitudes was used in [26] to estimate the change in the proportion of beta structures in the protein composition. In this study, we chose the Amide I/Amide II amplitude ratio as an indicator of the proportion of alpha structures in the composition of hemoglobin.

**Figure 9:**
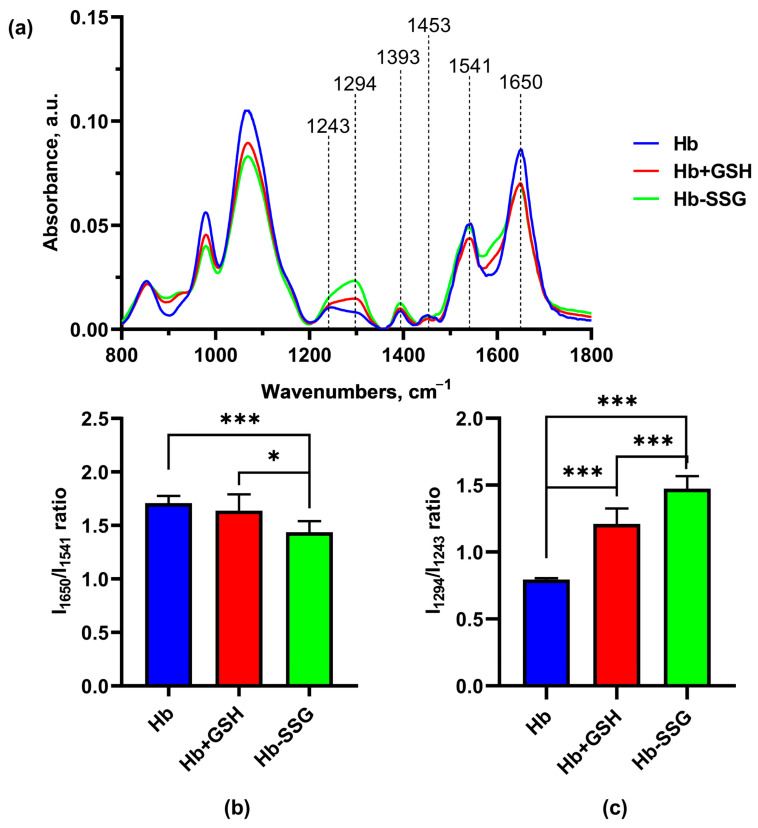
Averaged FTIR spectra (**a**) of hemoglobin oxyHb (blue), oxyHb + GSH (red), and oxyHb-SSG (green). (**b**) Intensity ratio of the I_1650_/I_1541_ bands in the FTIR spectra. (**c**) Intensity ratio of the I_1294_/I_1243_ bands of the FTIR spectra. n = 8. * *p* < 0.05, *** *p* < 0.001.

It was found that the IR spectra of Hb were characterized by a decrease (*p* < 0.001) in the I_1650_/I_1541_ ratio for Hb-SSG relative to the control, indicating a decrease in the proportion of alpha helices in the protein molecule (Figure 9b). In the Amide III region, a shift in the maximum from 1243 cm^−1^ in the control to 1294 cm^−1^ in the Hb + GSH and Hb-SSG samples was observed. The I_1294_/I_1243_ ratio increased (*p* < 0.001) in the spectrum of the Hb + GSH complex and, to an even greater extent, in the spectrum of Hb-SSG (Figure 9c). It was shown earlier that three characteristic sites characterizing the type of protein secondary structure were present in the Amide III region. The band intensities from 1320 cm^−1^ to 1295 cm^−1^ reflect the content of alpha helices in the secondary structure. The section from 1295 cm^−1^ to 1255 cm^−1^ corresponds to a disordered structure, and the intensity of the bands from 1254 cm^−1^ characterize the representation of beta layers in the protein molecule [27]. Thus, the formation of the Hb + GSH complex and glutathionylation of Hb increase the proportion of disordered structure in the protein molecule, which is most pronounced in the case of glutathionylated hemoglobin.

## 3. Discussion

In erythrocytes, the main low-molecular-weight thiol of mammalian cells, glutathione, does not only play an important antioxidant role and protect proteins from irreversible oxidation, but also actively interacts with hemoglobin and changes its properties. The covalent binding of hemoglobin to glutathione—glutathionylation, which increases under oxidative [6] and metabolic stress [9]—leads to an increase in oxygen affinity [28]. The formation of a noncovalent complex of hemoglobin with glutathione allows erythrocytes to release glutathione during hypoxia, which enhances the antioxidant capacity of cells [3]. The formation of a complex with glutathione also increases the oxygen affinity of hemoglobin, but this increase is less significant than after glutathionylation [3].

In this study, changes in the hemoglobin molecule resulting from the formation of a noncovalent complex of hemoglobin with reduced glutathione, as well as during hemoglobin glutathionylation, are characterized. Since the crystal structures of these substances have not been obtained so far, the question of changes in the structure of hemoglobin during the formation of the corresponding compounds remains open.

An increase in the affinity of hemoglobin for oxygen can be caused by a change in the position of the heme, since it is iron in the composition of the heme that directly coordinates oxygen during binding [1]. Changes in the heme environment can be monitored using circular dichroism spectra in the region of the Soret band. The heme environment was first studied using this method in 1969 [29]. It was shown [29] that the circular dichroism spectrum, which reflects the position of heme and its state, is sensitive to the oxygen saturation of hemoglobin and to the degree of iron oxidation in the heme composition. The position of the oxyhemoglobin maximum in the circular dichroism spectrum shifts to the shorter wavelength region and has a lower amplitude compared to the maximum of deoxyhemoglobin. The maximum in the absorption spectrum of metHb (Fe^3+^) is located in an even shorter wavelength region compared to oxyHb. The absorption spectra of various forms of hemoglobin in the Soret region are characterized by the same maximum distribution as in the case of circular dichroism spectra.

We found that a noncovalent complex of hemoglobin with GSH leads to a shift in the peak and a decrease in the amplitude of the peak in the spectra of circular dichroism and absorption in the region of the Soret band. This demonstrates a change in the heme environment due to conformational rearrangements during the formation of the complex. According to the literature data, a decrease in the amplitude of these peaks indicates contact with the ligand, and the shift in the peak to the short-wavelength region may be due to the exposition of the heme group from the crevices to the exterior part of the subunit [30,31]. Raman data indicate a change in the conformation of the porphyrin macrocycle, namely, a change in the region of side radicals of hemoporphyrin and a decrease in the contribution of symmetric bending vibrations of pyrrole rings, which indicates a change in the protein environment of hemoporphyrin. It should be noted that in this case, hemoglobin was in the oxy form and the ratio of hemoglobin forms (oxyHb, deoxyHb, and metHb) did not change during the experiments. It follows from the analysis of absorption spectra in the long-wavelength region (500–620 nm) and a comparison of the characteristic peaks [32] for different forms of hemoglobin (Supplementary, Figure S1). Accordingly, the observed changes were not caused by a change in the ratio of different forms of hemoglobin. The formation of the Hb complex with GSH did not lead to a change in the secondary structure of the hemoglobin molecule according to CD data in the ultraviolet region. However, IR spectroscopy data indicate the presence of some change in the secondary structure, namely, an increase in the proportion of disordered structure in the protein molecule. A decrease in the melting temperature of hemoglobin in a complex with GSH indicates a lower thermal stability of the quaternary structure of the protein, which may be a consequence of a change in the three-dimensional structure of the protein that occurs as a result of a conformational transition or due to a partial disruption of the interaction between parts of the protein molecule, which may lead to some “loosening” of the structure. The Raman scattering data characterizing the change in the conformation of the Hb heme molecule confirm this assumption. Indeed, crystalline hemoglobin in a complex with glutathione does not have enhancement for the 665 nm band (at 633 nm excitation) characteristic of hemoglobin. As noted in the literature, such an increase is observed only in cells that normally contain hemoglobin at a concentration close to the solubility limit, and in crystalline Hb samples, but not in Hb solution. Therefore, it is assumed that this enhancement is the result of long-range exciton interactions generated by the superposition of exciton transitions at a high heme concentration [19]. It should be noted that the crystalline sample is closest in Raman spectrum characteristics to concentrated cellular hemoglobin. Our data indicate that the dense packing of hemoglobin in complex with glutathione changes, probably becomes less dense.

Previously, we found [3] the binding sites of reduced glutathione with hemoglobin using the method of molecular modeling. According to these data, the Trp37 beta subunit enters the third and fourth sites in the oxyhemoglobin. At the same time, it was shown [14] that Trp37 makes the main contribution to the tryptophan fluorescence of hemoglobin, while hemoglobin contains a total of six tryptophans (three in each αβ dimer—α14 Trp, β15Trp, and β37Trp). Trp37 is located at the interface between alpha-beta dimers (α1β2 interface) and, accordingly, its fluorescence intensity is sensitive to conformational changes in hemoglobin [14]. We found that the formation of a noncovalent complex of hemoglobin with GSH leads to a significant increase in the intensity of tryptophan fluorescence of the protein, presumably due to complexation and conformational changes in the protein that result in tryptophan displacement to the more hydrophobic environment. Indeed, according to the data of isothermal titration calorimetry, the formation of a complex of Hb with GSH is an entropy-favorable process, which indicates the removal of hydrophobic residues from the solvent [3]. According to the literature data, the intensity of tryptophan fluorescence is increased in a hydrophobic environment [32]. This assumption is also confirmed by the data of molecular modeling, which showed that, during the formation of a noncovalent complex, Trp37 becomes significantly less exposed to the solvent, while the availability of other tryptophan residues does not change (Figure 5). Another factor that possibly increases tryptophan fluorescence is the positional shift of Trp37 from heme, consequently weakening the nonradiative transitions between heme and tryptophan [33]. It is interesting to note that the mutation of βTrp37 leads to an increased affinity of hemoglobin to oxygen and a decrease in cooperativity that indicates a significant role of the βTrp37 residue in the formation of the oxygen binding site [34]. Thus, upon the noncovalent binding of reduced glutathione, the environment of Trp37 and its position change, which may consequently increase the oxygen affinity. According to our modeling data [3], one of the pairs of noncovalently bound glutathione is located near the heme of the beta subunit. However, unlike glutathione covalently bound to Cys112, that is located directly in the alpha helix of the beta subunit, noncovalently bound glutathione does not directly disrupt the alpha-helical sites. The change in the heme environment is accompanied by a change in tertiary and quaternary structure of hemoglobin.

Unlike the noncovalent complex of hemoglobin with GSH, which can be formed under normal conditions, Hb glutathionylation increases under conditions of oxidative [6] and metabolic stress [9].

The quaternary structure of the deoxy form Hb contains salt bridges at the subunit interface, which were first identified by Perutz [1]. In this case, the binding of oxygen to the heme iron and its further displacement leads to the displacement of the F helix and the destruction of salt bridges. The data obtained using NMR [10] show that glutathionylation destroys the corresponding bridges (βAsp94-βHis146 and hydrogen bond βAsp99-aTyr42) at the subunit interface, which converts hemoglobin into an oxy-like conformation. There is an opinion that the absence of salt bridges increases the affinity for oxygen, since oxygen binding demands the destruction of salt bridges that require the additional energy.

Hemoglobin contains six cysteine residues, one each in the alpha subunits (Cys104) and two each in the beta subunits (Cys93 and Cys112). Currently, glutathionylation at Cys93β [35], and Cys112β [28] residues has been proven, while data on Cys-104α glutathionylation are contradictory [3,36].

The hemoglobin region containing Cys93 was studied in [11] using the hydrogen/deuterium exchange method. It was found that glutathionylation led to a slight increase in the conformational flexibility of the peptide containing Cys93. The increased flexibility of the glutathionylated site can be caused by the disappearance of numerous noncovalent interactions at the αβ interface, which results in a decrease in cooperativity [11]. In order to find out the effect of glutathionylation on the intramolecular position of the heme, in the immediate vicinity of which an alpha helix containing Cys93 is present, the spectrum of circular dichroism in the region of the Soret band was recorded. We found no changes in the peak position. However, the decrease in alpha-helicity and the increase in disordered structures during the glutathionylation of hemoglobin were revealed using the CD method, proving an increase in the friability of alpha helices during the glutathionylation of hemoglobin. These data were also confirmed using infrared spectroscopy. A decrease in the intensity ratio of the Amide I to Amide II bands indicates a decrease in the percentage of alpha-helicity of glutathionylated hemoglobin. At the same time, the thermal stability of the protein remains unchanged. The thermostability of the protein depends not only on the secondary structure, but also on the stability of the tertiary and quaternary structure of the protein. Despite the changes in the secondary structure, the tertiary and quaternary structure of the glutathionylated Hb does not change significantly, as evidenced by the absence of changes in tryptophan fluorescence, heme position, and changes in thermostability. It should also be noted that the registration of protein melting by the Nanotemper Tycho NT.6 device is carried out by tryptophan fluorescence, which is mainly determined by the stability of the tertiary–quaternary structure of the protein, but not its secondary structure.

Currently, there is no comprehensive information on structural changes in the region of glutathionylated hemoglobin containing Trp37. However, according to the data obtained by the hydrogen/deuterium exchange method, glutathionylation of the oxy form of hemoglobin leads to an increase in conformational flexibility in this region of the molecule [11]. At the same time, we showed that the glutathionylation of hemoglobin does not change the intensity of fluorescence, the main contribution to which is made by Trp37. Thus, the environment of tryptophan does not change, although, according to the literature, the conformational flexibility of the site containing Trp37 increases. An important role in the interpretation of the obtained results is played by the understanding of an exact cysteine residue that can be glutathionylated in oxyhemoglobin, that is difficult to predict now.

Since the information on the possible glutathionylation of Cys104 is controversial and, according to our modeling data, Cys104 is not available for the solvent, for glutathionylation [3], the main candidates are Cys93 and Cys112. However, unlike Cys112, which is always available for glutathionylation, the Cys93 residue is available only in deoxyhemoglobin [3]. So, the glutathionylation of this cysteine may be conformationally dependent. In this way, it is a so-called cryptic cysteine, as is the case with some titin cysteines [37]. If we assume that Cys112 is mainly glutathionylated in the oxyhemoglobin, it can explain the absence of an effect of glutathionylation on the heme environment and the observed changes in the secondary structure, since Cys93 is located close to the heme, unlike Cys112, which is in the alpha helix of the beta subunit far from the heme.

The glutathionylation of hemoglobin increases in a number of pathologies [6]. Normally, about 4% of hemoglobin in erythrocytes is the glutathionylated, and the maximum glutathionylation increase—about 69%—was registered in diabetes mellitus with microangiopathy [4]. There are also data on increased protein glutathionylation during smoking [36]. Hemoglobin obtained from the blood of smoking donors was studied using the CD method and fluorometric method [38]. However, some of the obtained results differ from our results. It is most likely due to the fact that our work was conducted on pure commercial protein after its reduction and gel filtration, while hemoglobin in [38] was obtained directly from the blood of donors and was not further purified in any way, and the purity or post-translational modifications of the samples were not characterized. With such sample preparation, there is a high risk that hemoglobin continues to be in a noncovalent complex with glutathione, the existence of which was shown recently [3]. Moreover, since the concentration of reduced glutathione in the blood of smokers is increased [39], such complexes in this case may be greater than in the control.

The obtained data are important for understanding the functioning of hemoglobin in erythrocytes, since the level of glutathionylated hemoglobin is significantly increased in various pathologies associated with the development of oxidative stress [4,6], and the noncovalent complex is important for the adaptation of erythrocytes to hypoxic conditions [3]. In addition, the influence of glutathione on the properties of extracellular hemoglobin, which is above normal levels in plasma in pathologies associated with hemoglobinemia [40], cannot be excluded. However, extracellular hemoglobin is rapidly oxidized to met-hemoglobin [40]. Because glutathione can reduce metal ions [41,42], it is possible to reduce methemoglobin with GSH. Moreover, the extracellular space and serum are more oxidizing environments than the intracellular medium. Blood plasma GSH content varies, averaging from 2 to 4 [43] up to several tens of µM (0.47 and 415 μM) [44]. According to our data, the dissociation constant of oxyHb with GSH is about 2 μM [3]. Thus, at high GSH content in blood plasma and locally increased concentration of extracellular hemoglobin, the formation of oxyHb-GSH complex is possible. In such a case, changes in the properties of extracellular hemoglobin can be expected. However, at low concentrations of glutathione and hemoglobin, their complexation probability would be low. Due to the oxidative environment of plasma and glutathionylation potential of methemoglobin, processes like the formation of glutathionylated hemoglobin or the met-Hb reduction by GSH are suggested to prevail in blood plasma.

Thus, it was found that the glutathionylation of oxyhemoglobin leads to a change in the secondary structure of the protein, reducing the proportion of alpha-helicity, but does not affect the heme environment, tryptophan fluorescence, or the thermal stability of the protein. During the formation of a noncovalent complex of hemoglobin with glutathione, the secondary structure remains almost unchanged; however, changes in the heme environment and the microenvironment of tryptophans, as well as a decrease in the protein’s thermal stability, are observed. Thus, covalent and noncovalent binding of glutathione to hemoglobin lead to various conformational changes. During the glutathionylation of oxyhemoglobin, despite the change in the secondary structure, there are no signs of a change in the tertiary and quaternary structure of hemoglobin, while the formation of a noncovalent complex has a significant effect on these structures.

## 4. Materials and Methods

### 4.1. Sample Preparation

Purified Hb solution was prepared as follows. Human metHb (Sigma-Aldrich, H7379, St. Louis, MO, USA) (0.1 mM) was dissolved in 50 mM K-phosphate buffer (50 mM KCl, 34.8 mM K_2_HPO_4_, 15.2 mM KH_2_PO_4_, 2 mM MgCl_2_, pH 7.4) and reduced using 5 mM sodium dithionite for 10 min. The solution with reduced Hb was passed through a gel filtration column (PD MiniTrap G-25, UK). Elution was performed using 50 mM K-phosphate buffer.

GSH (AppliChem, A2084, Germany) (4 mM) was allowed to interact with oxyHb to form a noncovalent complex, as described in [3]. Oxy-Hb was incubated with oxidized glutathione (GSSG) (AppliChem, A2243, Germany) (4 mM) for two hours at room temperature to obtain glutathionylated hemoglobin.

### 4.2. Circular Dichroism (CD) Studies

CD spectra were recorded on a Jasco J715 spectropolarimeter (Japan) at 20 °C. Two sets of wavelength ranges, 190–250 nm and 300–500 nm, were chosen for these studies. The 190–250 nm range was used to monitor the secondary structure. Hb samples at a concentration of 0.45 mg/mL were placed in quartz cuvettes of path length 1mm. The other range (300–500 nm), that spans the Soret region, was used for the detection of the structural alterations caused by heme. Quartz cuvettes of path length 1cm were used and the protein concentration was 0.3 mg/mL. All spectra shown are averages of 2 scans recorded at a scan rate of 100 nm/min. The spectra were corrected by subtracting appropriate buffer blanks. The measured UV spectra were resolved into components representing different types of secondary structure and their relative percentages were calculated using the program CDNN [45].

### 4.3. Absorption Spectrum Measurements

Absorption spectra were recorded on Jasco v-550 spectrophotometer (Japan) in the range of 400–620 nm to detect the Hb states (oxyHb, deoxyHb, and metHb). Quartz cuvettes of path length 1cm were used and the scan rate was 100 nm/min. We estimated the efficiency of metHb reduction with sodium dithionite (Na₂S₂O₄) using the algorithm first proposed by [13]. The method uses the first derivative of the spectrum at 645 nm and allows the determination of the metHb saturation percentage and the total hemoglobin concentration in the sample.

### 4.4. Fluorescence Spectroscopy Measurements

The spectra of Hb-SSG and the noncovalent Hb + GSH complex, at a concentration of 14 μM, were recorded from 305 to 450 nm on a Cary Eclipse fluorometer (USA) with quartz cuvettes (path length 1 cm). Trp fluorescence was measured using 298 nm excitation [14], excluding the effect of tyrosines. Excitation/emission bandwidths were 5 nm each, with a scanning rate of 120 nm/min. The spectra were corrected by subtracting appropriate buffer blanks and smoothed using a Savitzki–Golay filter in Matplotlib.

### 4.5. Thermal Stability Measurement

Nanotemper Tycho NT.6 (Germany) was used to measure the thermal stability of Hb-SSG and the noncovalent Hb + GSH complex. Hb at a concentration of 0.07 mM was placed in a volume of 10 µL in capillaries for measurements.

### 4.6. Western Blotting Assay

The effectiveness of hemoglobin S-glutathionylation was assessed using Western blot analysis. After the incubation of hemoglobin with GSSG, a Tris-Glycine SDS sample buffer (“Novex”, Carlsbad, CA, USA) was added without mercaptoethanol. Tris-glycine electrophoresis was performed in 14% of PAAG. Before incubation in a blocking buffer (5% milk), the membranes were fixed with a 5% formalin solution for 40 min to reduce the loss of alpha and beta Hb subunits (12–13 kDa). Primary mouse Anti-Glutathione antibody (“Millipore”, MAB5310 1:1000, Temecula, CA, USA) was used for the detection of S-glutathionylated proteins and rabbit anti-human Recombinant Anti-Hemoglobin subunit beta/ba1 antibody (ab92492, Abcam 1:1000, Germany) for the detection of Hb.

### 4.7. Raman Spectroscopy Measurement

The Raman scattering spectra of the sample were recorded using a combined confocal microscope–Integra Spectrum spectrometer (NT-MDT, Zelenograd, Russia). The registration parameters were as follows: laser, 633; power attenuation, 0.4ND; grating, 600; pre-exposure time to reduce fluorescence, 20 s; and spectrum registration time, 10 or 20 s. The Hb samples were dried as a drop on a mirror glass, and the spectra were recorded from the edge of the drop.

The spectra were processed in the Spectragraph v program.1.2. The spectra were corrected by subtracting the baseline using the built-in algorithm; then, we normalized the spectra for the total area under the curve and determined the intensities of the exact bands.

### 4.8. IR Spectroscopy Measurement

The infrared spectra of the IR Hb sample were recorded with a Spectrum Two FT-IR (Perkin Elmer, USA) IR spectrometer with attenuated total internal reflection infrared spectroscopy and internal reflection (ATR-FTIR). A drop of sample solutions in a buffer was dried on a slide and pressed against a zinc selenide window. The signal was recorded in the range of 550–4000 cm^−1^ with a scanning rate of 4 cm^−1^ 4 times, and 4 spectra were measured from each drop. The obtained spectra were processed in Spectragraph 1.2. The spectra were represented as a dependence of absorption on the wave number.

### 4.9. Modeling

The model of oxyhemoglobin in complex with 4 glutathione molecules construction was described in a previously published paper [3]. The structure of oxyhemoglobin (1R1X) used for model construction was obtained from Protein Data Bank (rcsb.org). The solvent-accessible surface area (SASA) was calculated for the oxyhemoglobin structure (1R1X) and for the model containing 4 glutathione molecules using the server GETAREA [17] (https://curie.utmb.edu/getarea.html (accessed on 22 August 2023)). SASA was calculated per individual residue using a probe with radius of 1.4 Å.

### 4.10. Statistics

Statistical analysis was performed using GraphPad Prism. The Shapiro–Wilk test was used for checking if the data were normally distributed. An analysis of variance (ANOVA) was performed with the Tukey test for multiple comparisons to investigate the differences in several groups. Statistical significance was established as a value of *p* < 0.05. Values are shown either as means ± standard deviations or as boxplots with the minimum, the maximum, the sample median, and the first and third quartiles. The difference between the peaks of CD and absorption spectra in three groups was investigated with the Kruskal–Wallis test and Dunn’s test for multiple comparisons. The number of experiments and the *p*-values for each experiment are shown in the figures and in their captions.

## 5. Conclusions

It was established that the covalent attachment of glutathione to oxyhemoglobin—glutathionylation, which is increased under oxidative stress—causes changes in the secondary structure of the protein, but does not lead to significant alterations in its tertiary and quaternary structure. The formation of a noncovalent complex of hemoglobin with GSH, which occurs under normal conditions, on the contrary, has almost no effect on the secondary structure, but causes changes in the heme environment, tryptophan fluorescence, and melting temperature, indicating significant changes in its tertiary and quaternary structure.

## Figures and Tables

**Table 1:** Solvent-accessible surface area for oxyhemoglobin (1R1X) in complex with four molecules of glutathione calculated by us using server GETAREA (https://curie.utmb.edu/getarea.html) [17].

Residue	Total	Apolar	Backbone	Sidechain	Ratio (%)	Availability In/Out
Complex of oxyhemoglobin with four GSH molecules						
Trp14.α1	141.50	134.76	7.48	134.02	59.7	Out
Trp15.β1	27.67	23.53	2.76	24.91	11.1	In
**Trp37.β1**	**18.91**	**18.12**	**0.93**	**17.97**	**8.0**	**In**
Trp14.α2	141.50	134.76	7.48	134.02	59.7	Out
Trp15.β2	27.67	13.53	2.76	24.91	11.1	In
**Trp37.β2**	**17.76**	**17.76**	**0.25**	**17.50**	**7.8**	**In**
Structure of oxyhemoglobin (1R1X)						
Trp14.α	141.51	134.77	7.48	134.03	59.7	Out
Trp15.β	27.67	23.53	2.76	24.91	11.1	In
**Trp37.β**	**138.37**	**134.70**	**14.98**	**123.38**	**54.9**	**Out**

The residue is determined as solvent-accessible (out) with ratio > 50%. SASA results for structure (1R1X) without GSH are marked in bold (here, we present only α and β subunits due to their complete symmetry). Data for Trp37.β are highlighted in bold.

## Data Availability

Not applicable.

## Supplementary Materials

The supporting information can be downloaded at: https://www.mdpi.com/article/10.3390/ijms241713557/s1.

## Abbreviations

GSH—reduced glutathione, GSSG—oxydized glutathione, Hb—hemoglobin, SaO_2_—oxygen saturation, UV—ultraviolet, SASA—solvent-accessible surface area, Hb-SSG—glutathionylated hemoglobin, Hb + GSH—noncovalent complex of glutathione with hemoglobin, FTIR—Fourier-Transform InfraRed, IR spectroscopy—infrared spectroscopy, CD—circular dichroism, ATR—attenuated total reflection, oxyHb—oxyhemoglobin, deoxyHb—deoxyhemoglobin, and metHb—methemoglobin.
