# Supplementary material for: Changes in Hemoglobin Properties in Complex with Glutathione and after Glutathionylation
Source: Int J Mol Sci. 2023 Aug 31;24(17):13557. doi: 10.3390/ijms241713557 (PMC10487563; doi:10.3390/ijms241713557)
Supplement: Supplementary file 1 [file ijms-24-13557-s001.zip › ijms-2562746-supplementary.pdf]

Supplementary

Changes of the Hemoglobin Properties in Complex with Glutathione and After Glutathionylation

Iu.D. Kuleshova, P.I. Zaripov, Yu.M. Poluektov, A.A Anashkina, D.N. Kaluzhny, E.Yu. Parshina,

G.V. Maksimov, V.A. Mitkevic, A.A. Makarov and I.Yu. Petrushanko

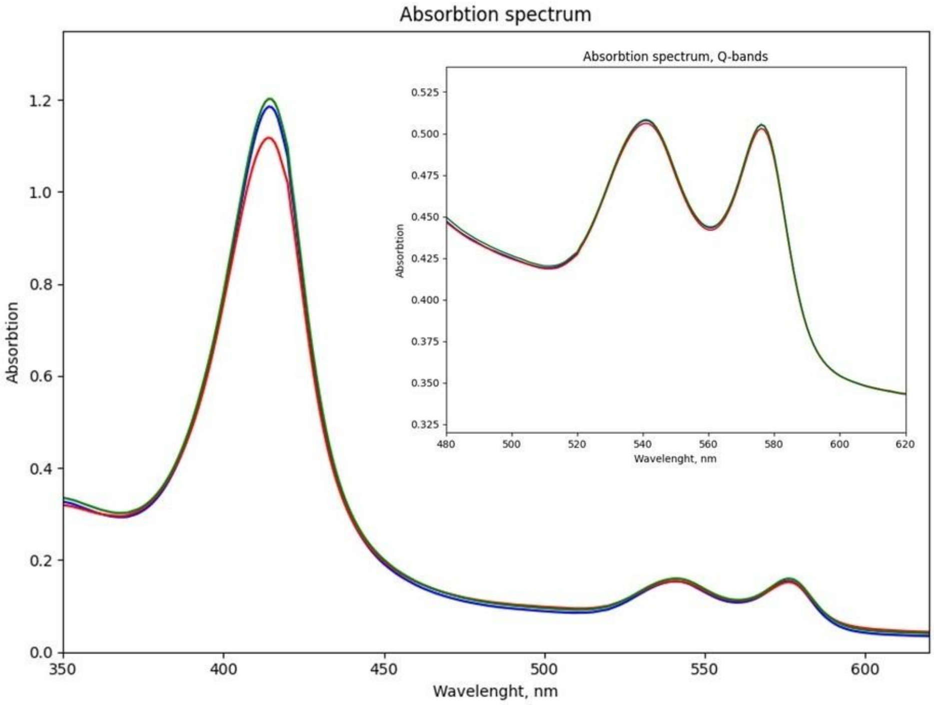

Figure S1. Absorption spectrum of hemoglobin (blue), glutathionylated hemoglobin (green) and non-covalent complex of hemoglobin with GSH (red).

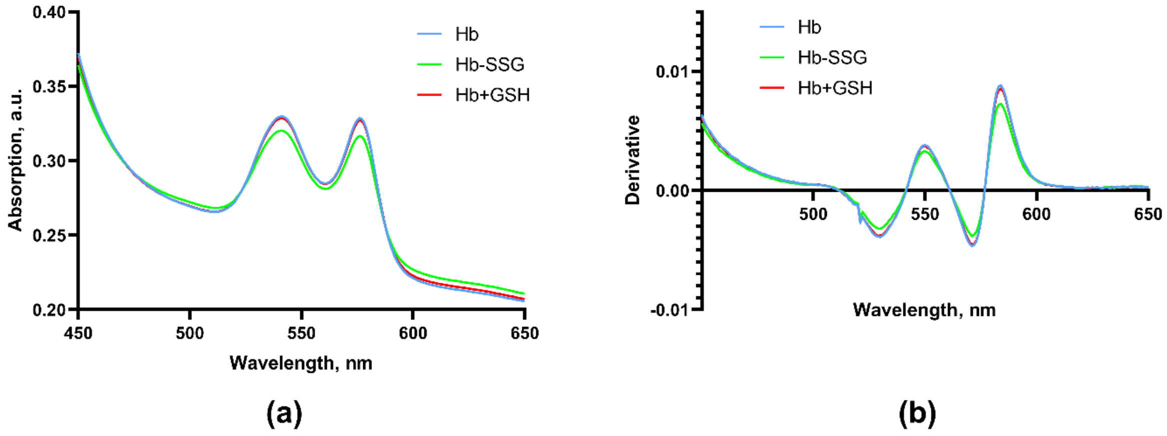

Figure S2. Efficiency of metHb reduction with sodium dithionite. (a) – absorption spectra of the samples; (b) – first derivative spectra of the samples. hemoglobin Blue – hemoglobin (Hb), green – glutathionylated hemoglobin (Hb-SSG), red – non-covalent complex of hemoglobin with GSH (Hb+GSH).

a

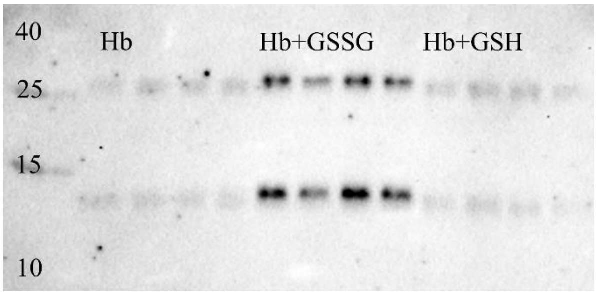

b

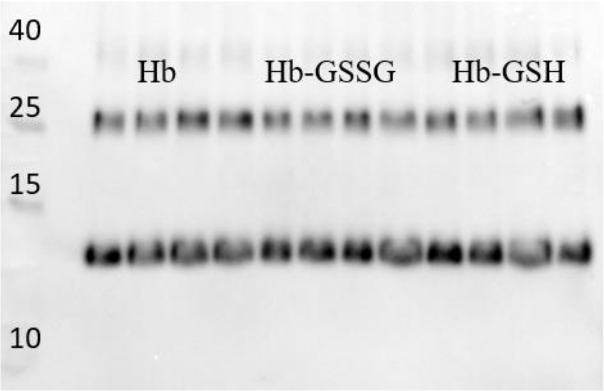

Figure S3. (a) – The original immunoblotting readouts, mouse monoclonal anti-glutathione antibody (a) and rabbit monoclonal anti-α Hb subunit antibody (b) were applied to detect glutathionylated proteins and total amount of α-subunit correspondingly. The upper bands correspond to hemoglobin dimers. Bottom bands - hemoglobin monomer. The bands corresponding to the monomers are used in the article for analysis (Fig. 1).
